# Interleukin Signatures as Prognostic Biomarkers in Ulcerative Colitis: From Immune Pathways to Clinical Prediction

**DOI:** 10.3390/cimb48020140

**Published:** 2026-01-27

**Authors:** Nikolaos Martinos, Andreas C. Lazaris, Christos Kroupis, Georgios Kranidiotis, Georgia-Eleni Thomopoulou

**Affiliations:** 1 Gastroenterology Department, Naval Hospital of Athens, 70 Dinokratous St., 11521 Athens, Greece; g.kranidiotis@gmail.com; 2 First Department of Pathology, School of Medicine, National and Kapodistrian University of Athens, 11527 Athens, Greece; 3 Department of Clinical Biochemistry, Attikon University General Hospital, National and Kapodistrian University of Athens, 12462 Athens, Greece; ckroupis@med.uoa.gr; 4 Cytopathology Department, Attikon University General Hospital, School of Medicine, National and Kapodistrian University of Athens, 12462 Athens, Greece; gthomop@med.uoa.gr

**Keywords:** ulcerative colitis, biomarkers, prognosis, histology, immune imbalance

## Abstract

Ulcerative colitis (UC) is a chronic immune-mediated inflammatory disease characterized by substantial heterogeneity in histologic activity, which is frequently uncoupled from clinical symptoms and endoscopic findings. Persistent microscopic inflammation is increasingly recognized as a critical determinant of relapse, therapeutic failure, and long-term disease outcomes, underscoring the need for molecular frameworks that align directly with tissue-level immune dysregulation. Interleukins (ILs) represent central regulators of mucosal immunity in UC, integrating innate and adaptive immune responses that govern epithelial injury and resolution. In this narrative review, we synthesize mechanistic, translational, genetic, and clinical evidence examining IL networks associated with histologic disease activity and persistence. Particular emphasis is placed on IL-23-driven inflammatory pathways, which consistently align with histologic severity, sustained microscopic inflammation, and resistance to immune resolution. In contrast, preserved IL-10-mediated regulatory signaling characterizes histologic remission and effective mucosal healing, whereas its insufficiency permits ongoing tissue-level inflammation. Downstream effector ILs, including IL-6, IL-1β, IL-8, and IL-17A, are discussed as mediators translating upstream immune imbalance into neutrophil recruitment and epithelial injury. Throughout this review, the term “prognostic” is used to denote alignment with histologic disease behavior rather than validated prediction of clinical outcomes. Collectively, the evidence supports the concept that coordinated IL patterns reflect distinct immunopathologic states underlying microscopic inflammation in UC, providing a biologically coherent framework for interpreting histologic activity and disease persistence beyond symptom-based assessment.

## 1. Introduction

Ulcerative colitis (UC) is a chronic immune-mediated inflammatory disease of the colon, characterized by relapsing-remitting mucosal inflammation arising from complex interactions between genetic susceptibility, environmental factors, the intestinal microbiota, and dysregulated host immune responses [1,2,3]. Despite substantial therapeutic advances and the availability of multiple biologic and small-molecule agents, UC remains a heterogeneous condition with marked variability in disease course, treatment response, and long-term outcomes [4,5].

A persistent challenge in UC management is the frequent discordance between clinical symptoms, endoscopic findings, and histologic inflammation. Accumulating evidence demonstrates that microscopic inflammatory activity may persist despite clinical remission or apparent endoscopic improvement and that histologic inflammation is more closely associated with relapse risk, corticosteroid dependence, hospitalization, and colorectal neoplasia than symptom-based or endoscopic indices alone [6,7,8,9]. These observations have shifted attention toward histology-aligned disease assessment and reinforced the need for biological frameworks that directly reflect tissue-level immune activity.

ILs play a central role in orchestrating mucosal immune responses in UC and represent key molecular mediators linking immune dysregulation to epithelial injury and repair. Coordinated interactions between innate and adaptive immune compartments shape the IL milieu within the inflamed colonic mucosa, influencing disease initiation, propagation, and resolution [10,11,12]. Within this complex network, specific IL pathways consistently align with microscopic disease activity, suggesting that histologic inflammation reflects structured immune states rather than nonspecific inflammatory noise.

Among these pathways, extensive experimental, translational, and clinical evidence implicates the IL-23 axis as a dominant driver of persistent mucosal inflammation in UC [13,14,15,16]. IL-23 sustains pathogenic immune activation, stabilizes inflammatory signaling circuits, and promotes downstream effector responses that contribute directly to epithelial injury and histologic damage [17,18,19,20]. Elevated circulating and mucosal IL-23 levels have been repeatedly associated with histologic severity, disease persistence, and therapeutic refractoriness, underscoring its close alignment with tissue-level inflammation [21,22,23,24]. Genetic associations involving the IL23R locus further support the biological relevance of this pathway in shaping inflammatory phenotypes across populations [25,26,27].

Counterbalancing pro-inflammatory signaling, IL-10 functions as a key regulatory cytokine essential for maintaining mucosal immune homeostasis. Produced by regulatory T cells, macrophages, and epithelial cells, IL-10 limits excessive immune activation, suppresses pro-inflammatory cytokine production, and supports epithelial integrity [28,29,30,31]. Experimental disruption of IL-10 signaling results in spontaneous intestinal inflammation, highlighting its non-redundant role in controlling mucosal immune responses [32].

In UC, however, IL-10-mediated regulation is frequently insufficient during active disease. Although IL-10 concentrations may increase during recovery or remission phases, multiple studies demonstrate reduced or functionally impaired IL-10 signaling during histologically active inflammation, particularly at the mucosal level [33,34,35,36]. This imbalance between heightened pro-inflammatory drive and inadequate regulatory counter-control contributes to sustained microscopic inflammation and incomplete immune resolution. The limited efficacy of recombinant IL-10 therapy further underscores the complexity of IL-10 biology and suggests that spatial, temporal, and signaling-context factors critically determine its regulatory impact [37,38].

Beyond IL-23 and IL-10, several downstream ILs—including IL-6, IL-1β, IL-8, and IL-17A—act as effector mediators translating upstream immune imbalance into neutrophil recruitment, crypt injury, and epithelial barrier disruption [39,40,41]. These cytokines correlate closely with histologic activity and relapse risk, reinforcing the concept that IL profiles mirror microscopic inflammatory burden more accurately than conventional systemic biomarkers such as C-reactive protein [42,43,44].

Collectively, these observations support a model in which histologic inflammation in UC arises from a dynamic imbalance between coordinated pro-inflammatory IL networks and insufficient regulatory control. In this narrative review, we synthesize mechanistic, translational, and clinical evidence to examine IL configurations aligned with histologic disease activity and persistence, with particular emphasis on IL-23-driven inflammatory pathways, IL-10-mediated regulation, and downstream effector ILs that translate immune dysregulation into microscopic tissue injury.

In contrast to prior studies that evaluated individual ILs as isolated biomarkers, the present review integrates IL networks into histology-aligned immune states. Within this framework, “prognosis” is not defined as prediction of discrete clinical events, but as the persistence or resolution of coordinated inflammatory and regulatory immune configurations at the tissue level. This state-based interpretation provides a biologically coherent link between histologic activity and disease behavior beyond symptom-based assessment.

## 2. Materials and Methods

This narrative review was designed to integrate mechanistic, translational, and clinical evidence on IL-mediated immune pathways in ulcerative colitis (UC), with particular emphasis on their prognostic relevance to histologic disease activity. A targeted, concept-driven approach was adopted, focusing on ILs with established biological plausibility and consistent associations with microscopic inflammation, disease persistence, and therapeutic outcomes.

A targeted literature search was conducted in MEDLINE (PubMed), EMBASE, and Web of Science, covering publications from January 1995 to March 2025. Search terms combined disease-related keywords (“ulcerative colitis”) with cytokine-related terms (“interleukin-23”, “interleukin-10”, “interleukin-6”, “interleukin-1β”, “interleukin-8”, “interleukin-17A”) and histology-related concepts (“histologic activity”, “histologic remission”, “Geboes”, “Nancy”, “Robarts”). Reference lists of relevant original articles and review papers were also manually screened to identify additional studies of interest.

Study inclusion was guided by relevance to the central narrative framework of this review. Priority was given to studies in adult patients with UC that reported quantitative assessment of ILs in serum, plasma, stool, or intestinal tissue using validated laboratory methods and linked these measurements to histologic disease activity, remission, relapse, or therapeutic response. Experimental and translational studies were considered when they provided mechanistic insights directly applicable to human UC pathophysiology.

Given the heterogeneity across study designs, cytokine assays, biological compartments, and histologic scoring systems, no attempt was made to quantitatively pool data or perform meta-analysis. Instead, evidence was synthesized narratively, emphasizing consistency of directionality, biological coherence, and translational relevance. Where available, representative quantitative findings were summarized to illustrate relative differences between disease states, while acknowledging methodological variability across studies.

ILs were organized into thematic axes reflecting their dominant immunological roles in UC: IL-23 as a central pro-inflammatory driver, IL-10 as a key regulatory cytokine, IL-6 and IL-1β as inflammatory amplifiers, and IL-8 and IL-17A as downstream effectors of tissue injury. This framework enabled the construction of integrated IL signatures aligned with histologic outcomes, supporting a biologically grounded approach to prognostic stratification in ulcerative colitis.

A total of 14 primary clinical studies evaluating IL profiles in relation to histologic endpoints in ulcerative colitis were included in this narrative review and are summarized in the accompanying tables.

Across these studies, cohort sizes ranged from small exploratory cohorts (*n* ≈ 20) to larger observational populations exceeding 300 patients, reflecting heterogeneity in study design and scale.

For clarity and consistency, the study numbers reported in the summary tables correspond directly to the numbering of references in the reference list.

## 3. Rationale and Contemporary Relevance of Histology-Aligned Immune Frameworks

Despite major advances in therapeutic options and the widespread adoption of treat-to-target strategies in ulcerative colitis (UC), a critical gap persists between clinical disease control and durable mucosal healing. This well-established discordance highlights the limitations of symptom- and endoscopy-based assessment and underscores the need for biologically grounded frameworks aligned with tissue-level immune activity. However, histology is still often interpreted as a static descriptive endpoint rather than as a biologically meaningful readout of underlying immune regulation. This disconnect has limited the integration of histologic assessment into mechanistic disease models and has constrained its interpretive value in both clinical practice and translational research.

At the same time, growing evidence indicates that persistent microscopic inflammation reflects stable and self-sustained immune configurations rather than residual or delayed inflammatory activity. Within this context, ILs represent central organizers of mucosal immune states, coordinating innate and adaptive responses that determine whether inflammation resolves or persists at the tissue level. Rather than functioning as isolated biomarkers, IL networks provide insight into the qualitative nature of immune dysregulation underlying histologic activity.

This evolving understanding creates a timely need for integrative frameworks that align histologic findings with biologically coherent immune mechanisms. By synthesizing mechanistic, translational, and clinical evidence, this review addresses this gap by examining how coordinated IL patterns correspond to distinct histologic immune states in UC. Framing histologic activity through this lens offers a unified approach to interpreting disease persistence and remission beyond conventional symptom-based or endoscopic assessment.

## 4. Immunopathogenesis of Ulcerative Colitis: IL Networks and Histologic Inflammation

Ulcerative colitis (UC) is characterized by a dysregulated mucosal immune response that results in sustained epithelial injury and chronic inflammation of the colonic mucosa [44,45]. Rather than reflecting a single dominant immune pathway, UC pathogenesis arises from a dynamic imbalance between pro-inflammatory signaling and insufficient regulatory control within the intestinal microenvironment [45,46]. ILs constitute central mediators of this imbalance, coordinating interactions between epithelial cells, antigen-presenting cells, innate immune populations, and CD4^+^ T-cell subsets.

Histologically active UC is defined by characteristic microscopic features, including crypt architectural distortion, crypt abscess formation, epithelial erosion, and dense infiltration of neutrophils and mononuclear cells within the lamina propria [47,48]. Importantly, histologic inflammation frequently persists despite clinical remission or apparent endoscopic healing, reflecting the persistence of coordinated immune dysregulation at the tissue level [49,50,51]. These observations underscore the biological relevance of histology as a disease dimension and highlight the need to identify molecular pathways that closely align with microscopic activity.

The mucosal cytokine milieu in UC reflects coordinated activation of innate and adaptive immune responses. Pro-inflammatory ILs—including IL-23, IL-6, IL-1β, IL-8, and IL-17A—are consistently upregulated in active disease and contribute to leukocyte recruitment, epithelial barrier disruption, and amplification of inflammatory signaling [52]. In parallel, regulatory cytokines, most notably IL-10, function to restrain immune activation and promote mucosal tolerance. The relative balance between these opposing forces appears to determine the persistence or resolution of histologic inflammation.

IL-10 represents a cornerstone of intestinal immune regulation. Early clinical studies demonstrated that circulating IL-10 levels increase during recovery phases of inflammatory bowel disease, whereas reduced IL-10 availability is associated with persistent inflammation [53,54]. These findings established defective IL-10-mediated regulation as a permissive factor for chronic mucosal inflammation rather than a compensatory response.

In contrast, IL-23 has emerged as a central pathogenic cytokine driving chronic intestinal inflammation. IL-23 is produced predominantly by antigen-presenting cells and tissue-infiltrating innate immune populations within the inflamed colonic mucosa and promotes the expansion and stabilization of pathogenic Th17 lymphocytes. Elevated mucosal and circulating IL-23 levels have been repeatedly associated with increased histologic severity, neutrophil infiltration, and crypt damage in UC [55,56]. Genetic studies further reinforce the relevance of this pathway, as polymorphisms in the IL23R gene confer susceptibility to UC and influence disease phenotype across populations.

The IL-23–Th17 axis contributes to sustained inflammation through downstream effector cytokines such as IL-17A and IL-22, which amplify neutrophil recruitment and epithelial injury [57,58]. Importantly, excessive IL-23 signaling also interferes with regulatory pathways by functionally antagonizing IL-10-mediated immune suppression, thereby reinforcing a self-perpetuating inflammatory loop. This reciprocal imbalance between IL-23-driven inflammation and IL-10-dependent regulation represents a defining immunopathologic feature of histologically active UC [59,60,61,62].

Beyond IL-23 and IL-10, additional ILs contribute to shaping microscopic inflammation. IL-6 and IL-1β promote leukocyte activation, epithelial apoptosis, and cytokine amplification and correlate closely with validated histologic indices such as the Geboes and Nancy scores [63,64,65,66]. IL-8, a potent neutrophil chemoattractant, mirrors crypt abscess formation and has been consistently associated with microscopic disease activity and impending relapse [67,68]. Together, these cytokines form integrated inflammatory networks that reflect histologic inflammation more accurately than clinical symptoms or systemic biomarkers alone.

Taken together, current evidence supports a pathogenesis-driven model in which histologic inflammation in UC arises from the convergence of excessive pro-inflammatory IL signaling—dominated by the IL-23 axis—and insufficient regulatory control mediated by IL-10. This imbalance provides a robust biological framework for interpreting microscopic disease activity and sets the foundation for the focused analysis of individual ILs and histology-aligned cytokine patterns in the following sections.

### 4.1. IL-23 and Histologic Inflammation in Ulcerative Colitis

IL-23 occupies a central position at the interface of innate and adaptive immunity, enabling amplification and stabilization of pathogenic inflammatory responses within the colonic mucosa.

Experimental evidence demonstrates that IL-23 can drive intestinal inflammation independently of IL-12, establishing it as an upstream regulator of pathogenic immune activation rather than a redundant inflammatory mediator. This distinction has been critical in defining the IL-23 axis as a central driver of sustained tissue-level inflammation in inflammatory bowel disease [69,70].

In ulcerative colitis (UC), elevated serum and mucosal IL-23 expression are consistently associated with histologic disease activity. Cross-sectional and case–control studies report significantly higher IL-23 levels in histologically active disease compared with histologic remission, with concentrations correlating with neutrophilic infiltration, crypt abscess formation, and epithelial injury. These associations are stronger with validated histologic indices, including the Geboes and Nancy scores, than with clinical activity measures, underscoring the close alignment of IL-23 with microscopic inflammation.

Mechanistically, IL-23 sustains the expansion and pathogenic stability of T helper 17 (Th17) lymphocytes, promoting downstream effector cytokine production, including IL-17A and IL-22. Activation of this axis enhances neutrophil recruitment, disrupts epithelial barrier integrity, and perpetuates crypt injury, thereby reinforcing histologic inflammation [71]. Consistent with these mechanisms, circulating and mucosal IL-23 levels increase with escalating histologic severity, with higher concentrations observed in moderate-to-severe microscopic disease compared with mild activity or remission, independent of disease duration.

Notably, IL-23 expression may remain elevated in the presence of persistent microscopic inflammation despite clinical improvement, indicating that IL-23 reflects tissue-level inflammatory burden rather than symptom-based disease activity. Longitudinal observations further associate sustained IL-23 elevation with ongoing histologic activity and reduced likelihood of achieving durable microscopic remission [72,73,74,75].

Genetic studies provide additional biological support for the pathogenic relevance of the IL-23 axis. Variants within the IL23R gene are associated with UC susceptibility and inflammatory phenotype, with risk alleles enhancing downstream cytokine signaling and protective variants attenuating inflammatory responses. Beyond its direct pro-inflammatory effects, excessive IL-23 signaling can functionally antagonize regulatory immune pathways, including IL-10-mediated suppression, thereby limiting effective resolution of mucosal inflammation. An overview of representative studies evaluating IL-23 in relation to histologic endpoints in UC is summarized in Table 1.

Collectively, these data establish IL-23 as a dominant upstream mediator tightly aligned with histologic inflammation in UC. By coordinating innate and adaptive immune activation and sustaining downstream effector pathways, IL-23 provides a mechanistic foundation for persistent microscopic disease activity, forming the biological basis for the histology-associated inflammatory patterns discussed in subsequent sections.

### 4.2. IL-10 and Failure of Immune Regulation in Ulcerative Colitis

IL-10 is a key anti-inflammatory cytokine that plays a non-redundant role in maintaining intestinal immune homeostasis and preventing excessive mucosal inflammation. IL-10 is produced by multiple immune and non-immune cell populations within the intestinal mucosa, including regulatory T cells, macrophages, dendritic cells, and epithelial cells, and exerts its effects by suppressing antigen presentation, inhibiting pro-inflammatory cytokine release, and promoting epithelial barrier repair [76].

The critical regulatory function of IL-10 in intestinal inflammation is underscored by experimental and translational studies demonstrating that disruption of IL-10 signaling leads to spontaneous colitis and uncontrolled mucosal immune activation. These observations established IL-10 as a central checkpoint in limiting pathogenic immune responses within the gut and highlighted its importance in preventing chronic tissue injury.

In human inflammatory bowel disease, early clinical studies showed that circulating IL-10 concentrations increase during recovery phases, whereas reduced IL-10 availability characterizes active inflammatory states. Subsequent investigations focusing on ulcerative colitis (UC) confirmed that impaired IL-10 signaling is associated with persistent histologic inflammation and failure to achieve sustained microscopic remission. These findings indicate that IL-10 insufficiency represents a permissive factor for ongoing mucosal inflammation rather than a mere epiphenomenon.

When disease activity is assessed at the histologic level, the relevance of IL-10 becomes particularly evident. Multiple cross-sectional and cohort studies have reported significantly lower serum or mucosal IL-10 levels in patients with histologically active UC compared with those in histologic remission, even in the absence of overt clinical symptoms. Reduced IL-10 expression has been associated with ongoing neutrophilic infiltration, crypt architectural distortion, and epithelial damage, underscoring its close alignment with microscopic disease activity.

Mechanistically, IL-10 constrains mucosal inflammation by suppressing the production of key pro-inflammatory ILs, including IL-23, IL-6, and IL-1β, by antigen-presenting cells and innate immune populations. Defective IL-10-mediated regulation permits sustained activation of pathogenic Th17 responses and amplification of downstream effector pathways, thereby perpetuating histologic inflammation. This regulatory failure appears particularly relevant in UC, where immune activation may persist despite partial clinical or endoscopic improvement [77].

Longitudinal evidence further supports the histology-aligned relevance of IL-10. Higher baseline serum or mucosal IL-10 levels have been associated with subsequent histologic improvement and sustained microscopic remission, whereas persistently reduced IL-10 expression characterizes patients at increased risk of histologic relapse despite apparent clinical quiescence. In this context, IL-10 reflects effective immune resolution rather than transient suppression of inflammatory activity.

The complexity of IL-10 biology in UC is further illustrated by the failure of recombinant IL-10 therapy to demonstrate consistent clinical efficacy in randomized trials, despite its clear anti-inflammatory properties. These results suggest that the regulatory effects of IL-10 depend on precise spatial, temporal, and receptor-level signaling dynamics that may not be adequately reproduced by systemic cytokine administration [78].

An overview of representative studies evaluating IL-10 in relation to histologic endpoints in UC is summarized in Table 2. Rather than defining a validated biomarker, these findings support IL-10 as a histology-aligned indicator of immune resolution and mucosal healing. When interpreted alongside excessive IL-23-driven inflammatory signaling, insufficient IL-10-mediated regulation emerges as a key mechanism underlying persistent microscopic inflammation and incomplete mucosal healing.

### 4.3. IL-8 and IL-17A in Neutrophil Recruitment and Persistent Microscopic Inflammation

IL-8 and IL-17A represent downstream effector cytokines that directly link upstream immune dysregulation to neutrophil recruitment, epithelial injury, and persistence of microscopic inflammation in ulcerative colitis (UC). In contrast to upstream regulatory or amplifying ILs, these mediators act predominantly at the tissue level, translating immune imbalance into histologic damage.

IL-8 is the principal neutrophil chemoattractant within the inflamed colonic mucosa and has demonstrated a strong quantitative association with neutrophilic infiltration, crypt abscess formation, epithelial erosion, and overall histologic severity. Multiple studies have reported increased mucosal and circulating IL-8 levels in patients with histologically active UC, with concentrations correlating closely with validated histologic indices, including the Geboes and Nancy scores. Importantly, IL-8 expression has been shown to remain elevated in patients with persistent microscopic inflammation despite clinical remission, highlighting its close alignment with tissue-level disease activity [79,80,81].

IL-17A, produced predominantly by Th17 lymphocytes and innate lymphoid cells, contributes to sustained neutrophil survival, cytokine amplification, and epithelial barrier disruption. Increased IL-17A expression has been consistently observed in actively inflamed colonic mucosa and correlates with crypt damage, neutrophil persistence, and higher histologic activity scores in UC. Notably, IL-17A levels may persist despite symptomatic improvement, providing a mechanistic explanation for ongoing microscopic inflammation in patients who appear clinically controlled.

Mechanistic studies have demonstrated functional cooperation between IL-17A and IL-8 within the inflamed mucosa. IL-17A induces IL-8 production by intestinal epithelial cells and stromal populations, thereby reinforcing neutrophil recruitment and perpetuating crypt injury. This feed-forward inflammatory loop stabilizes neutrophil-dominated inflammation and limits spontaneous histologic resolution [82,83].

Representative studies evaluating IL-8 and IL-17A in relation to primary histologic endpoints, including validated histologic activity scores and neutrophilic infiltration, are summarized in Table 3.

Collectively, IL-8 and IL-17A emerge as critical mediators of neutrophil-driven microscopic inflammation in UC. Their sustained expression during phases of apparent clinical remission underscores their role in maintaining subclinical tissue injury and explains, in part, the frequent discordance between clinical symptoms and histologic disease activity. When interpreted within the broader context of IL-23-driven inflammation and insufficient IL-10-mediated regulation, these effector cytokines complete a coherent immunopathogenic framework linking immune imbalance to persistent histologic inflammation.

### 4.4. Integrated IL Patterns Associated with Histologic Activity in Ulcerative Colitis

Although individual ILs provide important mechanistic insight, histologic activity in ulcerative colitis (UC) is more accurately reflected by the integrated balance between pro-inflammatory drivers, regulatory mechanisms, and downstream effector pathways. Accumulating evidence indicates that reproducible IL patterns, rather than isolated cytokine alterations, align most closely with microscopic disease activity and histologic outcomes.

Across studies examining mucosal and circulating cytokine expression, histologically active UC consistently exhibits a convergent immune configuration characterized by upstream inflammatory amplification, insufficient regulatory control, and sustained effector activity. Within this framework, dominant pro-inflammatory signaling—exemplified by IL-23-associated pathways—coexists with impaired counter-regulatory capacity and persistent expression of downstream mediators linked to neutrophil recruitment and epithelial injury. This coordinated imbalance stabilizes inflammatory immune states that are detectable at the tissue level.

Such integrated cytokine configurations provide a biological explanation for the frequent discordance observed between clinical or endoscopic improvement and ongoing microscopic inflammation. Partial attenuation of inflammatory signaling may reduce overt disease manifestations, while persistent effector activity and regulatory insufficiency maintain histologic abnormalities detectable on biopsy. In this context, histologic activity reflects the persistence of coordinated immune states rather than residual or nonspecific inflammation.

Conversely, histologic remission is associated with coordinated attenuation of inflammatory amplification and restoration of effective regulatory balance. Studies evaluating cytokine expression during microscopic healing demonstrate reduced expression of pro-inflammatory ILs alongside preserved or enhanced regulatory signaling, consistent with epithelial repair and resolution of neutrophilic infiltration. These observations support the concept that histologic healing represents an active and regulated immunological process.

Importantly, these IL patterns have been observed across different biological compartments, including serum and intestinal mucosa, and across diverse study designs, supporting their biological robustness despite methodological heterogeneity. Although absolute cytokine concentrations vary between studies, the directionality and internal coherence of these immune configurations remain consistent.

Rather than defining a validated cytokine “signature,” the available evidence supports the existence of integrated IL patterns that reflect distinct immunopathologic states aligned with histologic activity in UC. Together, these coordinated features define a reproducible histology-aligned immune state in ulcerative colitis.

### 4.5. Histologic Activity as a Stable Immune State Rather than a Transient Disease Phase

Histologic activity in ulcerative colitis (UC) has traditionally been interpreted as a downstream reflection of clinical disease severity or as a residual manifestation of incomplete therapeutic response. Within this framework, microscopic inflammation is often viewed as a delayed or passive consequence of overt disease activity, expected to resolve following clinical or endoscopic improvement.

However, accumulating evidence challenges this linear interpretation. Persistent histologic inflammation frequently occurs despite sustained clinical remission and apparent endoscopic healing, indicating that microscopic activity does not simply trail behind symptomatic control. Instead, histologic inflammation appears to reflect a stable and self-maintained immune configuration that can persist independently of clinical disease expression. Conceptualizing histologic activity as an immune state provides a unifying framework for interpreting why tissue-level inflammation may persist even when clinical indices or endoscopic appearance improve.

Partial modulation of inflammatory pathways may be sufficient to alleviate symptoms or improve mucosal appearance, while the underlying immune equilibrium that sustains microscopic inflammation remains largely intact.

Importantly, this state-based interpretation shifts the role of histology from a descriptive endpoint to a biologically meaningful readout of immune regulation at the tissue level. Histologic activity, in this context, reflects the persistence of coordinated immune signaling networks rather than the magnitude of inflammatory burden alone. Transitions between histologic activity and remission are therefore better understood as shifts between distinct immunological equilibria rather than as linear progression along a single disease continuum.

This reframing establishes histologic assessment as a window into immune state identity and provides the conceptual foundation for distinguishing pro-inflammatory and regulatory histology-aligned immune configurations, which are explored in the following sections.

### 4.6. Histologic Remission as an Actively Maintained Regulatory Immune State

Histologic remission in ulcerative colitis (UC) should not be interpreted as the passive consequence of inflammatory suppression or as a simple reduction in cytokine activity. Instead, accumulating evidence indicates that microscopic healing reflects the establishment of an actively maintained regulatory immune state, characterized by coordinated immune restraint and preservation of mucosal homeostasis.

Within this regulatory configuration, IL-10 functions not merely as an anti-inflammatory mediator, but as a central organizing axis that stabilizes immune equilibrium at the tissue level. Effective IL-10-mediated regulation limits excessive activation of innate and adaptive immune compartments, constrains downstream effector pathways, and permits epithelial repair without inducing global immunosuppression.

Importantly, the regulatory immune state associated with histologic remission is qualitatively distinct from partial inflammatory attenuation. Clinical or endoscopic improvement may occur in the absence of sufficient regulatory dominance, allowing low-grade effector activity to persist and maintain microscopic abnormalities. In contrast, durable histologic remission emerges when regulatory control predominates, suppressing pathogenic immune circuits and preventing re-establishment of inflammatory equilibria [84].

Longitudinal observations support this state-based interpretation, demonstrating that preservation of regulatory signaling is associated with sustained microscopic healing, whereas erosion of regulatory control precedes histologic relapse, even in patients who remain clinically asymptomatic. These transitions are best understood as shifts between regulatory and inflammatory immune states rather than as gradual fluctuations in inflammatory intensity.

Conceptualizing histologic remission as an actively maintained regulatory state provides a biological explanation for its strong association with favorable long-term outcomes and establishes a mechanistic counterpoint to pro-inflammatory immune configurations that sustain persistent microscopic inflammation.

### 4.7. Clinical Implications of Histology-Aligned IL Patterns in Ulcerative Colitis

Recognizing histologic activity in ulcerative colitis (UC) as the manifestation of coordinated immune states has important implications for how disease behavior is interpreted, rather than for how it is immediately managed. Clinical symptoms, endoscopic appearance, and systemic inflammatory markers reflect partial and often compartmentalized aspects of disease activity, whereas histologic assessment captures the persistence or resolution of immune configurations operating at the tissue level [84,85,86].

Within this framework, the frequent discordance between clinical remission and ongoing microscopic inflammation can be understood as a mismatch between symptomatic control and underlying immune state identity. Therapeutic interventions may attenuate upstream inflammatory signaling sufficiently to reduce symptoms, while downstream effector pathways and regulatory insufficiency continue to sustain histologic abnormalities. In such cases, apparent clinical stability may coexist with persistent immune imbalance at the mucosal interface.

Conversely, histologic remission reflects a qualitative shift toward a regulatory-dominant immune state in which inflammatory amplification is effectively contained. Importantly, this state cannot be reliably inferred from clinical indices alone. The normalization of histology-aligned immune configurations, rather than symptomatic improvement per se, appears to underpin durable microscopic healing and protection from relapse.

These observations underscore the interpretive value of histology-aligned IL patterns as biological correlates of disease behavior rather than as standalone predictive biomarkers. Their relevance lies in contextualizing histologic findings within coherent immunopathologic states, thereby clarifying why similar clinical presentations may be associated with divergent long-term outcomes.

**Figure 1:**
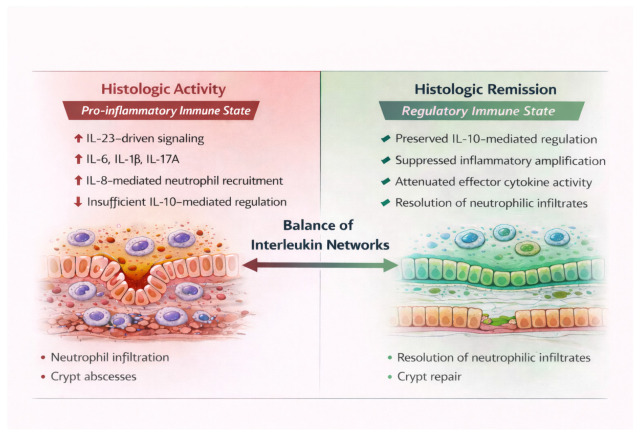
Histology-Aligned IL Immune States in Ulcerative colitis.

**Figure 2:**
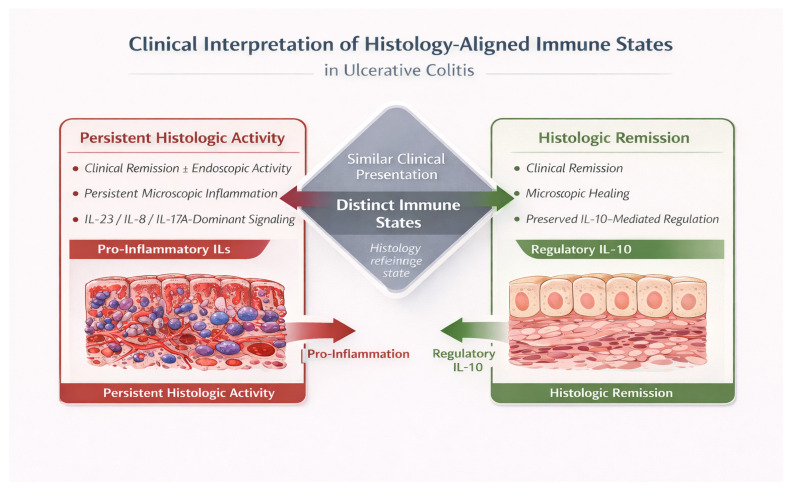
Clinical interpretation of histology-aligned immune states in ulcerative colitis.

Framing clinical disease behavior through histology-centered immune states provides a biologically grounded lens for integrating molecular mechanisms with tissue pathology. This perspective establishes a conceptual bridge between immunopathogenesis and histologic assessment, setting the stage for future longitudinal studies aimed at defining how transitions between immune states relate to disease persistence and resolution. These coordinated IL configurations define reproducible histology-aligned immune states in ulcerative colitis, as schematically illustrated in Figure 1. A schematic overview of the clinical interpretation of histology-aligned immune states is provided in Figure 2.

Conceptual overview of histology-aligned immune states in ulcerative colitis, illustrating the balance between inflammatory amplification and regulatory control across disease states. Arrows indicate directional and functional relationships between immune pathways and cytokine-mediated interactions.

This figure illustrates how histologic assessment reflects distinct underlying immune states in ulcerative colitis. Despite similar clinical presentation, patients may exhibit persistent histologic activity driven by dominant pro-inflammatory IL networks or achieve histologic remission characterized by effective regulatory immune control. This framework explains the frequent discordance between clinical findings and microscopic inflammation.

## 5. Limitations and Future Perspectives

### 5.1. Methodological Limitations

The present review is subject to several methodological limitations inherent to the available literature. The included studies display considerable heterogeneity with respect to study design, patient selection, disease activity definitions, and IL measurement techniques. Histologic assessment was performed using different scoring systems across studies, limiting direct quantitative comparisons and synthesis of results. Furthermore, most data derive from cross-sectional analyses, restricting the ability to capture dynamic immune state transitions over time or establish temporal relationships between immunologic patterns and histologic outcomes.

Prospective studies employing standardized histologic scoring systems, harmonized biomarker assays, and longitudinal sampling strategies will be essential to validate immune state dynamics and improve comparability across cohorts.

### 5.2. Conceptual Limitations

From a conceptual perspective, the immune state framework proposed in this review represents an integrative model based on associative evidence rather than a formally validated classification system. The delineation of inflammatory and regulatory immune states reflects coordinated IL patterns aligned with histologic findings and should not be interpreted as discrete, static, or mutually exclusive biological entities. Moreover, immune states likely exist along a continuum and may vary in stability depending on disease context, treatment exposure, and host-specific factors.

Prospective validation studies integrating immunologic, histologic, and clinical endpoints are required to refine immune state definitions, assess their temporal stability and reversibility, and determine their biological boundaries.

### 5.3. Translational Limitations

From a translational standpoint, the immune states described herein should not be regarded as immediate clinical decision-making tools. Currently, no validated biomarker thresholds, composite scores, or predictive algorithms exist to guide treatment escalation, de-escalation, or therapeutic selection based solely on IL profiles or histology-aligned immune states. Consequently, the proposed framework is intended to support biological interpretation rather than to function as a standalone prognostic or therapeutic instrument.

Future translational research integrating immune state assessment into interventional and longitudinal clinical trials may clarify how histology-aligned immune states can inform risk stratification, treatment monitoring, and personalized therapeutic strategies.

## 6. Conclusions

Histologic activity represents a biologically distinct and clinically relevant dimension of ulcerative colitis (UC), frequently persisting despite apparent clinical or endoscopic improvement. Accumulating evidence indicates that this microscopic inflammation reflects coordinated immune states rather than random or residual disease activity, underscoring the need for biological frameworks that align directly with tissue-level pathology.

This review synthesizes mechanistic, translational, and clinical data demonstrating that histologically active UC is characterized by reproducible IL network configurations. A pro-inflammatory pattern dominated by IL-23-driven signaling emerges as a central feature of persistent microscopic inflammation, integrating upstream immune activation with downstream effector mechanisms that sustain neutrophil recruitment and epithelial injury. In contrast, histologic remission aligns with restoration of effective regulatory control, most prominently mediated by IL-10, highlighting that mucosal healing represents an active and coordinated immunological process rather than the mere absence of inflammatory stimuli.

These IL patterns provide a mechanistic explanation for the frequently observed discordance between clinical symptoms and histologic findings. Partial suppression of inflammatory pathways may lead to symptomatic improvement, while persistence of histology-aligned immune configurations permits ongoing tissue-level injury. In this context, IL networks offer biological coherence to heterogeneous disease courses and variable long-term outcomes among patients with similar clinical presentations.

Importantly, the consistent association between specific IL configurations and histologic disease states suggests that ILs may have potential utility as biological indicators of microscopic inflammatory activity. Rather than functioning as standalone biomarkers or direct predictors of clinical outcomes, IL patterns may contribute to contextual interpretation of histologic findings, aid in identifying persistent immune activation despite clinical quiescence, and support a more mechanistically informed assessment of disease behavior.

The term “prognostic,” as used herein, therefore refers to alignment with histologic disease persistence and resolution rather than validated clinical outcome prediction. Within this framework, IL networks are best viewed as biological correlates of tissue-level immune states, with potential relevance for refining disease stratification and for informing future research on monitoring microscopic inflammation.

Integrating histology-centered immune frameworks with longitudinal IL assessment in future studies may clarify their role in tracking disease trajectories and distinguishing transient inflammatory suppression from durable immune resolution. A deeper understanding of IL network balance has the potential to enhance interpretation of histologic activity and to support more precise, mechanism-based approaches to disease assessment in ulcerative colitis. Framing UC as a disease of persistent immune states rather than episodic inflammation may ultimately redefine how microscopic activity is interpreted and monitored.

Importantly, the findings synthesized in this review support a state-based interpretation of ulcerative colitis, in which histologic activity and remission reflect coordinated inflammatory and regulatory immune states rather than transient disease phases. Within this framework, IL networks define stable histology-aligned immune configurations that underlie persistent microscopic inflammation or durable histologic remission. Reinforcing the concept of immune states in this manner provides a biologically coherent lens through which histology can be interpreted beyond clinical symptoms alone.

## Figures and Tables

**Table 1:** IL-23-Related Primary Histology-Aligned Prognostic Endpoints in Ulcerative Colitis.

Study	Design	Biomarker Source	Histology Endpoint	Main Finding	Histology-Aligned Interpretation
[8]	Cross-sectional	Serum	Histologic severity	Serum IL-23 levels were higher in patients with increased histologic severity.	Elevated IL-23 reflects dominance of a pro-inflammatory immune state associated with sustained microscopic activity and neutrophil-driven tissue injury.
[11]	Case–control	Serum	Histologic activity	Serum IL-23 concentrations were increased in histologically active UC compared with inactive disease.	Increased IL-23 indicates persistent upstream inflammatory signaling aligned with an active histologic immune configuration rather than transient inflammation.
[10]	Cross-sectional	Serum	Histologic activity	IL-23 levels increased with greater histologic severity and longer disease duration.	Progressive elevation of IL-23 supports its role in stabilizing chronic inflammatory immune states underlying persistent histologic activity.
[41]	Prospective	Serum	Histologic severity	Elevated baseline serum IL-23 levels were associated with subsequent severe histologic disease.	Baseline IL-23 elevation identifies a pre-existing inflammatory immune state predisposing to persistent or progressive microscopic inflammation.

Abbreviations: UC, ulcerative colitis; IL, IL-23.

**Table 2:** IL-10-Related Primary Histology-Aligned Prognostic Endpoints in Ulcerative Colitis.

Study	Design	Biomarker Source	Histologic Endpoint	Main Finding	Histology-Aligned Interpretation
[1]	Cross-sectional	Serum	Histologic inactivity	Serum IL-10 levels were higher in histologically inactive disease compared with active inflammation.	Increased IL-10 reflects dominance of a regulatory immune state associated with suppression of microscopic inflammation and histologic quiescence.
[3]	Prospective	Serum	Histologic improvement	Higher baseline serum IL-10 levels were associated with subsequent histologic improvement.	Preserved IL-10 signaling indicates effective regulatory immune capacity favoring transition toward histologic remission.
[62]	Cohort	Serum	Histologic remission	IL-10 concentrations increased during histologic remission compared with active disease	Elevation of IL-10 characterizes restoration of regulatory immune balance accompanying microscopic healing.
[4]	Case–control	Serum	Microscopic inflammation	Reduced serum IL-10 levels were observed in patients with histologically active UC.	Reduced IL-10 reflects insufficient regulatory control permitting persistence of inflammatory immune states at the tissue level.
[55]	Prospective	Mucosal	Sustained histologic remission	Higher mucosal IL-10 expression was associated with maintenance of histologic remission.	Sustained mucosal IL-10 expression supports long-term stabilization of a regulatory immune state underlying durable microscopic healing.

Abbreviations: UC, ulcerative colitis; IL, IL-10.

**Table 3:** Primary Histology-Aligned Prognostic Endpoints for IL-8 and IL-17A in Ulcerative Colitis.

Study	Design	Biomarker	Biomarker Source	Histologic Endpoint	Main Finding	Main Finding
[50]	Prospective	IL-8	Mucosal	Histologic activity	Mucosal IL-8 expression was increased in histologically active UC compared with histologic remission.	Increased IL-8 reflects a neutrophil-dominant inflammatory immune state driving crypt abscess formation and persistent microscopic activity.
[59]	Cross-sectional	IL-8	Mucosal	Histologic severity	IL-8 levels increased with greater histologic severity.	Progressive IL-8 elevation indicates amplification of neutrophil recruitment proportional to the intensity of histologic inflammation.
[71]	Cross-sectional	IL-8	Serum	Nancy index	Higher serum IL-8 levels were observed in patients with histologic activity compared with inactivity.	Elevated circulating IL-8 mirrors ongoing tissue-level neutrophilic inflammation despite potential clinical quiescence.
[28]	Cross-sectional	IL-17A	Serum	Geboes score	Serum IL-17A levels were higher in histologically active disease.	Increased IL-17A reflects sustained Th17-driven effector activity stabilizing inflammatory immune states at the microscopic level.
[45]	Cross-sectional	IL-17A	Mucosal	Nancy index	Mucosal IL-17A expression correlated with severe histologic activity.	Enhanced mucosal IL-17A supports persistence of neutrophil-survival signals and limits spontaneous histologic resolution.

Abbreviations: UC, ulcerative colitis; IL, IL-17A, IL-8.

## Data Availability

No new data were created or analyzed in this study. Data sharing is not applicable to this article.
